# Smart Automatic Irrigation Enhances Sap Flow, Growth, and Water Use Efficiency in Containerized *Prunus* × *yedoensis* Matsum. Seedling

**DOI:** 10.3390/plants13233270

**Published:** 2024-11-21

**Authors:** Eon-Ju Jin, Myung-Suk Choi, Hyeok Lee, Eun-Ji Bae, Do-Hyun Kim, Jun-Hyuck Yoon

**Affiliations:** 1 Forest Biomaterials Research Center, National Institute of Forest Science, Jinju 52817, Republic of Korea; jinej85@korea.kr (E.-J.J.); gosorock@korea.kr (E.-J.B.); 2 Division of Forest Environmental Resources, Institute of Agriculture and Life Science, Gyeongsang National University, Jinju 52828, Republic of Korea; mschoi@gnu.ac.kr (M.-S.C.); danny9605@naver.com (D.-H.K.); 3 Division of Forest Ecological Restoration, Baekdudaegan National Arboretum, Bonghwa 36209, Republic of Korea; hyeok0902@koagi.or.kr

**Keywords:** *Prunus* × *yedoensis* Matsum., smart automatic irrigation, container nursery, sap flow, chlorophyll fluorescence, photosynthetic ability

## Abstract

This study conducted a comparative analysis on the effects of smart automatic and semi-automatic irrigation methods on the physiological characteristics and growth of *Prunus* × *yedoensis* Matsum. seedlings. The smart automatic irrigation system, which activates irrigation when the soil moisture drops below 15%, demonstrated superior characteristics in sap-wood area and bark ratio, as well as excellent water management efficiency, compared to the semi-automatic irrigation method, which involves watering (2.0 L) for 10 min at 60 min intervals starting at 8 AM every day. The analysis of soil moisture content changes under varying weather conditions and irrigation methods showed that smart automatic irrigation effectively maintained optimal moisture levels. Moreover, sap flow in the smart automatic irrigation treatment was more efficiently regulated in response to seasonal variations, showing a strong correlation with climatic factors such as temperature and solar radiation. In contrast, the semi-automatic irrigation treatment led to excessive sap flow during the summer due to a fixed watering schedule, resulting in unnecessary water supply. Analysis of photosynthesis parameters and chlorophyll fluorescence also revealed that smart automatic irrigation achieved higher values in light compensation and saturation points, maximizing photosynthetic efficiency. These findings suggest that the smart automatic irrigation system can enhance plant growth and water use efficiency, contributing to sustainable water management strategies. This research provides critical foundational data for developing efficient agricultural and horticultural irrigation management strategies in response to future climate change.

## 1. Introduction

In 2019, there were a total of 8.25 million street trees planted across South Korea, with *Prunus* × *yedoensis* Matsum. trees accounting for 0.99 million, or 12% of the total [1]. Among the cherry tree genus, the Yoshino cherry (*Prunus* × *yedoensis*) is highly valued as an ornamental street tree and is the most widely used species across the country. It holds significant value, particularly during spring flower festivals, where its blossoms contribute greatly to the event’s appeal. The timing and quality of the blossoms are the most crucial factors for the success of cherry blossom festivals, as noted by Jung et al. [2]. Despite their popularity nationwide for their beautiful flowering characteristics, there is limited understanding of their water absorption and seasonal water requirements.

In nurseries, irrigation quantities are crucial for producing standardized, healthy seedlings [3]. According to the U.S. literature, the maximum irrigation requirement for a 25-year-old cherry tree is between 760 and 1000 mm [4]. Szűcs [5] suggested that a water supply equivalent to 700 mm rainfall would be optimal for cherry cultivation. Cherry trees are typically irrigated in spring, especially during flowering, to avoid water shortages [6].

Container seedling cultivation significantly impacts tree growth due to environmental factors such as moisture, light, and temperature, which are crucial in restricted pot volumes [7,8]. In landscaping nurseries, irrigation practices (frequency, timing, duration) are often based on personal experience, lacking quantitative methods to measure tree water needs, resulting in trial and error [9].

Accurate data are essential for maintaining optimal growth conditions, yet objective and scientific data on the relationship between irrigation quantities, frequency, rainfall, soil moisture, and the permanent wilting point of tree species are scarce. Plant transpiration, accounting for 80–90% of total evapotranspiration, is driven passively by negative water potential created by evaporation from leaf cells [10]. Sap flow measurement using heat pulse velocity is widely used due to its low cost, reliability, and low power requirements, providing estimates of whole-tree transpiration [11,12,13,14,15,16]. The Sap Flow Meter (SFM1) has been demonstrated to accurately and reliably measure zero, low, and high sap flow across a wide range of plant species under various environmental conditions following a short heat pulse [17].

The correlation between sap flow and meteorological parameters has been attempted in several studies, and sap flow has been found to have a positive correlation with air temperature [6,18]. A coefficient of determination (R^2^) of 0.73 indicated a strong positive linear correlation between the daily sap flow of olive trees and potential transpiration [19]. Additionally, while air and soil temperatures were highly correlated with relative changes in sap flow in red oak trees, active radiation, vapor pressure deficit (VPD), relative humidity, and photosynthetic activity had relatively small effects on sap flow rates [20].

According to the WMO’s annual report, there is an almost 80% chance that the global average temperature for one of the next five years (2024–2028) will be 1.5 °C higher than pre-industrial levels. Rising temperatures are expected to alter terrestrial water dynamics, indicative of regional aridity through differences in precipitation and potential evapotranspiration [21]. Understanding plant physiological responses is essential for assessing plant resilience to extreme weather conditions [22]. Sap flow measurements integrate various tree characteristics, including growth rate and conductivity, which serve as indicators of plant health [23]. Determining the optimal daily sap flow timing provides insights into individual trees’ optimal climatic conditions [22].

This study aims to estimate water usage in cherry trees (*Prunus* × *yedoensis*) subjected to different irrigation methods. Over ten months, we investigated water usage in container-grown trees under various irrigation treatments, aiming to provide optimal irrigation practices and quantities. By measuring sap flow, this study compared tree growth and physiological characteristics, analyzing responses to microclimate and soil moisture changes to develop indicators for irrigation management.

## 2. Materials and Methods

### 2.1. Experimental Stages

This study was designed to compare the effects of smart irrigation and semi-automatic irrigation on *Prunus* × *yedoensis* in Jinju, Gyeongsangnam-do, and to derive optimal irrigation management indicators. The experiment was conducted on eight rows of trees over an area of 0.3 ha. Smart irrigation was automatically activated when soil moisture fell below 15%, while semi-automatic irrigation operated daily at 8 AM for 10 min every 60 min.

Data collection was conducted using three main methods. First, sap flow analysis was performed using the SFM1 device at 20-min intervals, and soil moisture was measured using teros 12, 5TE, and Hydra probes. Second, growth rate measurements were carried out in March, June, and September, analyzing both absolute and relative growth rates. Third, physiological characteristics were analyzed through photosynthetic response measurements using a Li-6400 device and chlorophyll fluorescence analysis using a Handy Cam.

**Figure 1:**
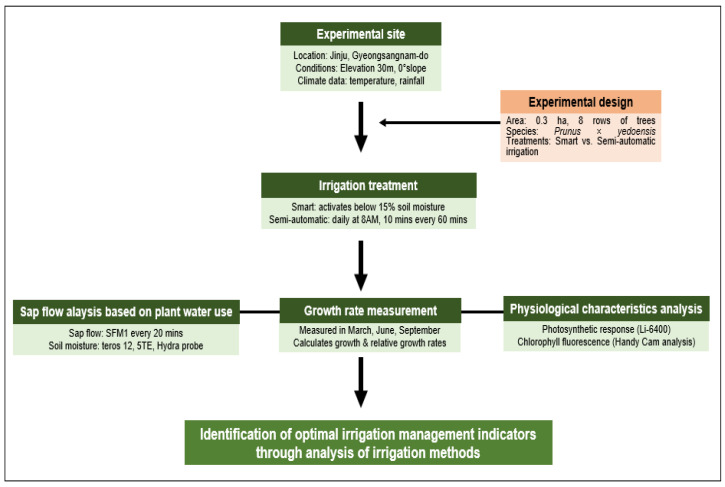
A schematic of a study to analyze water use, growth rate, and physiological characteristics of *Prunus* × *yedoensis* under various irrigation methods to develop optimal irrigation management indicators.

Based on these analyses, the effects of irrigation methods on the growth and physiological characteristics of cherry trees were evaluated, leading to the derivation of optimal irrigation management strategies (Figure 1).

### 2.2. Expermental Sites

**Figure 2:**
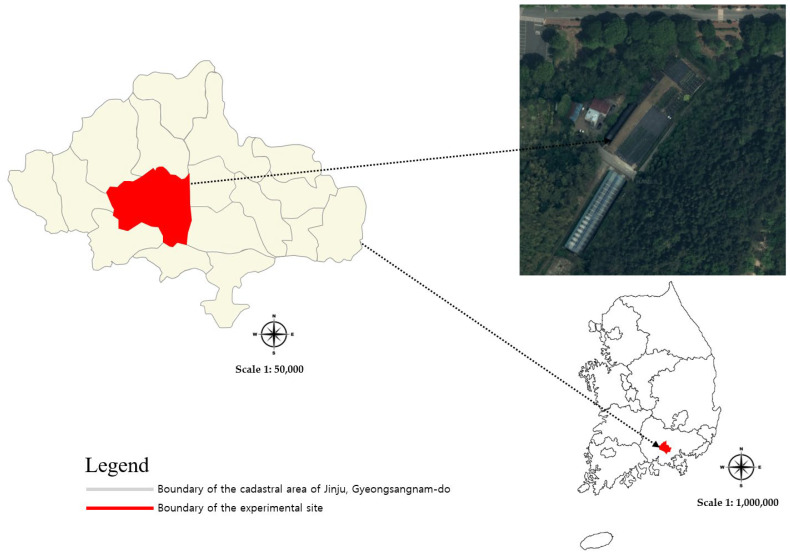
Location of the experimental site.

The experiment was conducted at the experimental forest of the Forest Biomaterials Research Center, National Institute of Forest Science, located in Jinju, Gyeongsangnam-do, South Korea (N35°09′48.5″, E128°06′03.6″) (Figure 2). The study area is situated at an elevation of 30 m with a slope of 0°. The region experiences a mild climate, and during the study period (1 March to 30 September 2023), the average temperature was 21.7 °C, with a maximum temperature of 36.4 °C and a minimum temperature of −1.0 °C. Total precipitation amounted to 331.9 mm, with July experiencing concentrated rainfall of 504.5 mm. Meteorological data (WP-700C, MIRAE SENSOR, Seoul, Republic of Korea) were collected at 10 min intervals, including measurements of temperature (°C), precipitation (mm), wind speed (m/s), humidity (%), vapor pressure (hPa), dew point temperature (°C), sunshine duration (h), solar radiation (MJ/m^2^), and ground temperature (°C).

### 2.3. Experimental Design

**Figure 3:**
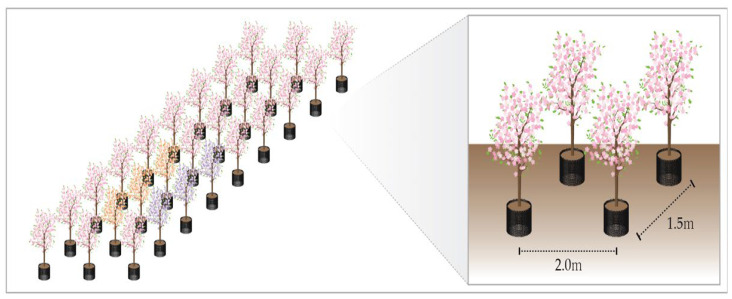
Experimental plot arrangement of *Prunus* × *yedoensis* for irrigation experiment. The study area covers 0.3 hectares, with *Prunus* × *yedoensis* trees of 4 cm diameter at breast height (DBH) arranged in 8 rows. The spacing between rows was 2.0 m, and the spacing between trees within a row was 1.5 m.

The study area covers 0.3 ha, and the trees are arranged in 8 rows. The spacing between rows is 2.0 m, and the spacing between trees within a row is 1.5 m (Figure 3). The test species used in the experiment is the Yoshino cherry tree (DBH 4 cm), which was grown at the National Institute of Forest Science’s Subtropical and Temperate Forest Research Institute. In March 2022, the trees were transplanted into container pots (40 L) filled with potting soil (7073926501, Gumok, Seoul, Republic of Korea) at the Experimental Forest of the Forest Biomaterials Research Institute of the National Institute of Forest Science and underwent an acclimatization process for one year. In March 2023, the height of the trees ranged from 3.1 to 3.3 m, and for the study, trees starting from the third row, where a weather station was installed, were selected. A total of six trees were divided into two groups for irrigation (smart automatic irrigation method and semi-automatic irrigation method), with each group consisting of three trees.

### 2.4. Irrigation Treatment

Irrigation was carried out using two methods: the smart automatic irrigation method and the semi-automatic irrigation method. The smart automatic irrigation system is an automated system that activates irrigation when the soil moisture content falls below 15%. The semi-automatic irrigation system involves a semi-automatic treatment where water (2.0 L) is applied for 10 min at 60 min intervals starting at 8 AM every day. In all treatment plots, drip irrigation was installed at a depth of 10 cm from the soil surface of the container pots.

### 2.5. Field Measurements

#### 2.5.1. Soil Moisture

Soil moisture content was monitored using Teros 12 (Meter Environment, Pullman, WA, USA), 5TE (Decagon, Pullman, WA, USA), and Hydra Probe (Stevens Water, Pullman, WA, USA) sensors. Measurements from the Teros 12 and 5TE sensors were obtained every 10 min using a ZL6 data logger (Meter Environment, Pullman, WA, USA), while the Hydra Probe recorded data every 10 min using a DT80 data logger (Data Taker, Thermo Fisher Scientific Pty Ltd., Seoul, Republic of Korea). The soil moisture sensors were installed at a depth of 5 cm from the surface layer of the container pots to observe changes in soil moisture content during the study period and to calculate volumetric moisture content.

#### 2.5.2. Sap Flow Measurement

**Figure 4:**
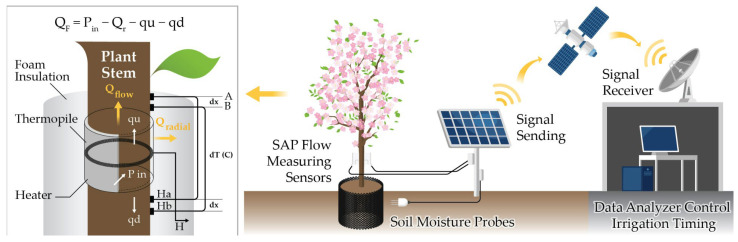
Sap flow measurement and irrigation control system schematic.

The Sap Flow Meter (SFM1), based on the Heat Ratio Method (HRM), was used to measure sap velocity and sap flow rates in the experimental trees (Figure 4). This method is highly reliable, requires minimal maintenance, and can measure zero, low, high, and reverse sap flows in various plant species [17,24]. The SFM1 consists of three measurement needles (one heater needle and two temperature needles), each 35 mm in length and 1.3 mm in diameter, which are independently connected to the device via cables. The temperature needles have two thermocouple junctions spaced 1.5 cm apart that measure the movement of heat released by the heater needle within the xylem tissue [22].

The SFM1 was installed at breast height on the stem of each tree for each irrigation treatment, after removing the bark. To enable continuous sap flow recording, the internal battery was connected to a specially designed power supply system consisting of two 12-volt batteries connected in series, which were regularly replaced. To protect the device from external heat sources like direct sunlight, the area around the sensor was wrapped with aluminum foil as a cover. Sap flow data were recorded every 20 min using a Campbell Scientific CR10 data logger.

### 2.6. Growth Rate

To assess growth, tree height and root collar diameter were measured repeatedly in March, June, and September 2023, as well as prior to irrigation treatment in early March 2022 after transplanting into container pots. These measurements were used to analyze growth and relative growth rates. The relative growth rate (RGR) for each unit period (day^−1^) and for the total growing period was calculated based on the initial measurements of height and root collar diameter before treatment [25].

RGR was calculated using Equation (1):

(1) $$RGR=\frac{\ln\left({D_{2}}^{2}-H_{2}\right)-\ln\left({D_{1}}^{2}-H_{1}\right)}{t_{2}-t_{1}}$$

- *D*_1_ and *H***_1_**: initial diameter and height at the beginning of the treatment;
- *D*_2_ and *H*_2_: diameter and height at the later measurement point (end of growth period);
- *t*_1_ and *t*_2_: time points of each measurement;
- ln: natural logarithm.

Additionally, the sturdiness quotient (H/D ratio: [SQ; Sturdiness Quotient = height (cm)/root collar diameter (mm)]), which is an indicator of the health and robustness of the grown trees, was calculated as the ratio of height (m) to root collar diameter (cm) [26].

### 2.7. Photosynthetic Responses

To investigate the effects of irrigation treatments on the photosynthetic characteristics of the study trees, healthy leaves were assessed using a portable photosynthesis system (Li-6400, LI-COR Inc., Lincoln, NE, USA). The light intensity during photosynthesis measurements was adjusted using an LED light source, allowing for control over Photosynthetic Photon Flux Density (PPFD) at nine levels: 0, 50, 100, 250, 500, 800, 1000, 1500, and 2000 μmol·m^−2^·s^−1^. These measurements were used to construct light-response curves. Photosynthetic parameters were calculated using values at a light intensity of 600 μmol·m^−2^·s^−1^, which matched the light intensity within the leaf chamber.

The airflow rate into the leaf chamber of the photosynthesis system was set at 500 μmol·s^−1^, with a temperature of 25 °C and relative humidity of 60%, minimizing the influence of external environmental changes. A CO_2_ injector system was attached to maintain a stable CO_2_ concentration within the range of 400 ± 2 μmol·mol^−1^, preventing rapid fluctuations.

The analysis of the light-response curves was based on the method of Kim and Lee [27], which was used to calculate dark respiration (Rd), light compensation point (LCP), light saturation point (LSP), maximum photosynthesis rate (PNmax), and apparent quantum yield (AQY). Stomatal conductance and transpiration rate were calculated using the formulas of von Caemmerer and Farquhar [28]. Water use efficiency (WUE) was assessed following the method of Kim et al. [29], using the photosynthesis rate (Pn) and transpiration rate (Tr) measured simultaneously (WUE = Pn/Tr).

### 2.8. Chlorophyll Fluorescence

Chlorophyll fluorescence analysis was conducted using a Handy Cam (FlorCam, Optimum Watering CZ, Průmyslová, Drásov, Czech Republic) through quenching kinetics analysis in a dark-adapted state (20 min of dark treatment in the chlorophyll fluorescence analyzer chamber), following the methods of Barbagallo et al. [30] and Genty et al. [31,32]. During measurements, chlorophyll fluorescence was induced using actinic light and a saturating light source. The analysis conditions for the Handy Cam were set with actinic light (red LED) at 200 μmol^−2^·s^−1^ and saturating light (moderate light) at 1250 μmol^−2^·s^−1^. The collected data were analyzed using the method proposed by Gorbe et al. [33] (Table 1).

## 3. Results

### 3.1. Characteristics of Seedlings Based on Irrigation Methods

**Figure 5:**
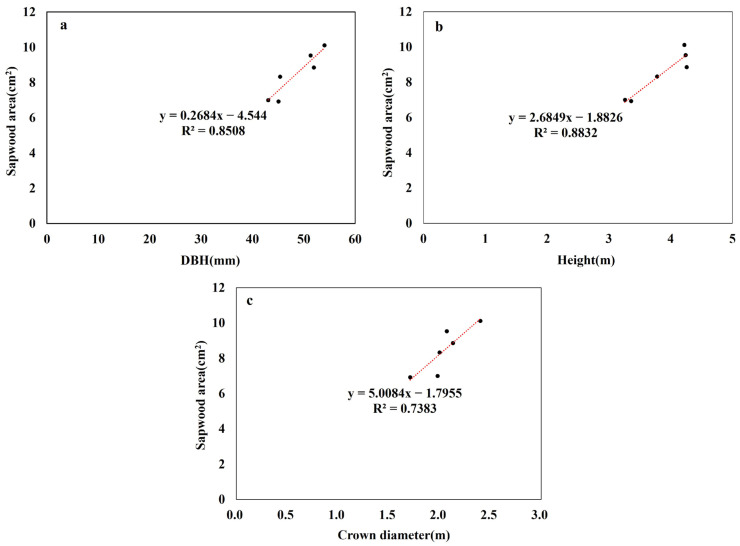
Correlations between DBH, height, and canopy area with sapwood area for the *Prunus* × *yedoensis* used in these experiments. (**a**) DBH and sapwood area; (**b**) height and sapwood area; (**c**) canopy area and sapwood area.

The wood characteristics of *Prunus* × *yedoensis* are presented in Table 2. The sapwood area of individual trees varied significantly depending on the irrigation method. In terms of bark ratio, the smart automatic irrigation treatment area showed a larger proportion compared to semi-automatic irrigation. When examining the Total Basal Area, sapwood accounted for over 60% of it. The coefficient of determination (R2) was used to correlate the sapwood area with diameter at breast height (DBH), tree height, and canopy area, revealing a strong positive linear relationship across these three data points, as illustrated in Figure 5.

### 3.2. Changes in Soil Moisture Content Based on Weather Conditions and Irrigation Methods

**Figure 6:**
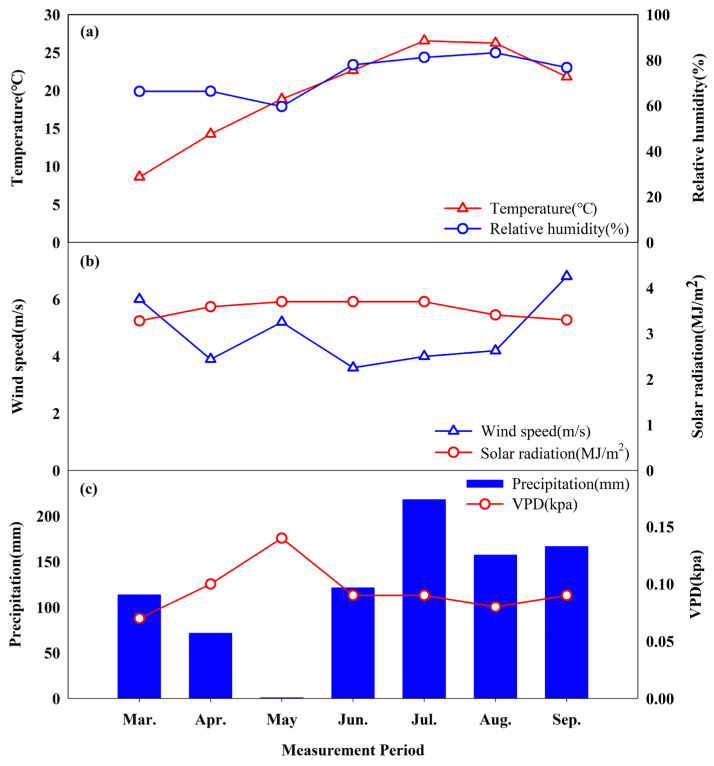
Meteorological parameters collected for the study sites. (**a**) Temperature and relative humidity; (**b**) wind speed and solar radiation; (**c**) precipitation and VPD.

Figure 6 presents the climatic parameters, including average temperature, relative humidity, wind speed, vapor pressure deficit (VPD), and precipitation, from January to October in the study area. These measured variables showed significant monthly variations. The monthly average temperatures in the study area were 3.4 °C in winter (December–February), 18.6 °C in spring (March–May), 25.2 °C in summer (June–August), and 20.0 °C in autumn (September–October). The total precipitation in May was only 0.9 mm, accounting for just 7.8% of the annual precipitation (871.6 mm), highlighting it as a dry month. The soil was driest in May due to the higher VPD and evapotranspiration rate, which are associated with lower relative humidity and higher temperatures.

**Figure 7:**
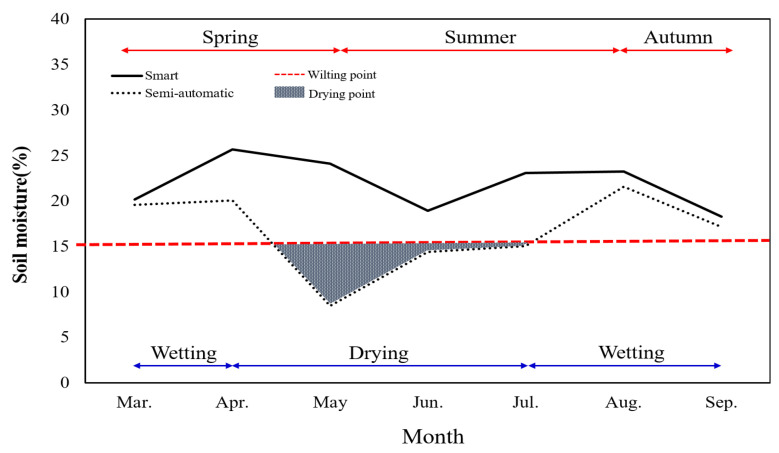
Soil moisture contents of the container pots.

Figure 7 shows the changes in soil moisture content within container pots over the 7-month monitoring period (1 March to 30 September) based on irrigation treatments. The monthly average temperature ranged from 18.3 to 25.7 °C with smart automatic irrigation and 8.5–21.6 °C with semi-automatic irrigation. According to previous research by the National Institute of Forest Science, the optimal soil moisture content range within containers for *Prunus* × *yedoensis* with a diameter at breast height (DBH) of 3 cm or more is 15–25% from March to April and 18–25% from May to August. In this study, smart automatic irrigation maintained optimal soil moisture content within the containers, while semi-automatic irrigation resulted in soil moisture levels below the recommended range.

Examining the monthly changes under different irrigation treatments, soil moisture content decreased from spring (March–April) to early summer (June) and increased again in July and August due to frequent rainfall and lower transpiration rates, before decreasing again in autumn (September). Throughout the study period, the research site generally remained wet due to substantial rainfall, with April and May being the only relatively dry months. Notably, May recorded rainfall below 1.0 mm, marking it as the driest month during the monitoring period.

### 3.3. Changes in Velocity and Sap Flow Based on Irrigation Treatments and Weather Conditions

**Figure 8:**
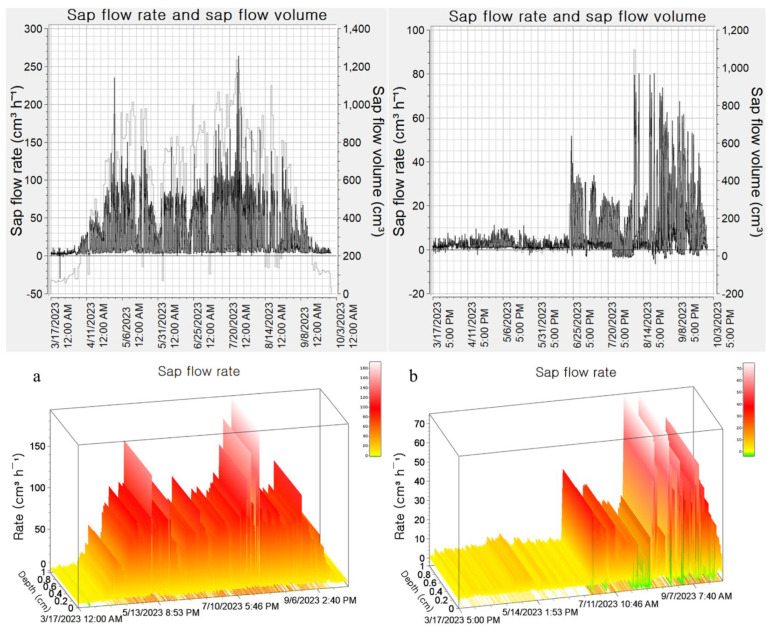
Daily changes in sap flow rate depending on irrigation treatment in container cultivation. (**a**) Smart automatic irrigation; (**b**) semi-automatic irrigation. The above figure represents the Raw Heat Pulse Velocity data in a 2D format, while the figure below depicts the 3D graphic representation of the original heat pulse velocity.

Figure 8 compares the flow velocity under different irrigation treatments from March to September 2023, showing significantly higher velocities with smart automatic irrigation compared to semi-automatic irrigation. In the smart automatic irrigation treatment, the flow velocity began to increase from 1 April, while in the semi-automatic treatment, it started to rise after 4 June.

**Figure 9:**
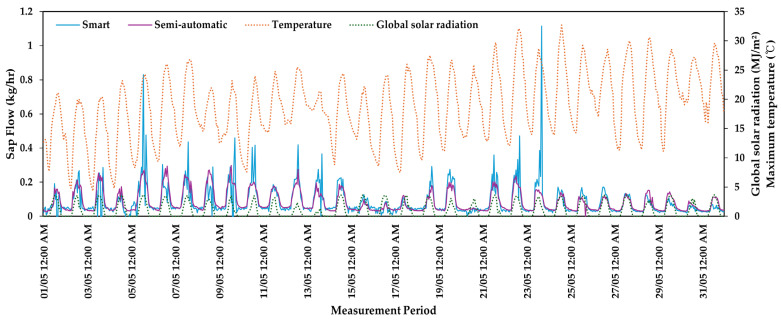
Comparison of sap flow rate in May for *Prunus* × *yedoensis* under different irrigation treatments in container cultivation against temperature and global solar radiation.

Figure 9 illustrates changes in sap flow during the driest month of May, comparing average temperature and global solar radiation. The sap flow mirrored temperature changes, showing a ‘bell-shaped’ curve: it gradually increased after sunrise (07:00), peaked around 1 PM, and then gradually decreased from 4 PM onward.

On 21 May, a notable decrease in sap flow was observed across all irrigation treatments, likely due to the low global solar radiation of 1.1 MJ/m^2^, which was significantly below the monthly average of 3.3 MJ/m^2^. There were four days in May (5th, 15th, 17th, 21st) when temperatures exceeded 30 °C.

### 3.4. Comparison of Monthly Sap Flow and Irrigation Volume

**Figure 10:**
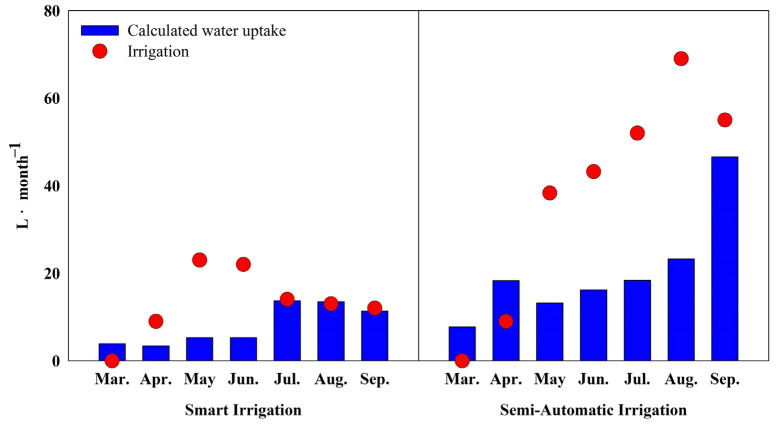
Comparison of cumulative water uptake in container-grown *Prunus* × *yedoensis* trees under different irrigation treatments: based on sap flow measurement and water supply.

The cumulative monthly transpiration and irrigation volumes for smart automatic irrigation and semi-automatic irrigation methods were analyzed (Figure 10). Sap flow volumes exhibited seasonal variations, changing with environmental conditions such as temperature and relative humidity. During spring (March–May), sap flow volumes were relatively low for both irrigation methods, aligning with the period of generally lower temperatures and reduced plant water demand. In the smart automatic irrigation method, the sap flow volume was 5.3 L in April, whereas the semi-automatic method recorded a particularly high flow of 18.3 L in May.

In summer (June–September), sap flow volumes significantly increased. The smart automatic irrigation method peaked at 13.7 L in July, showing moderate growth. In contrast, the semi-automatic method consistently recorded higher sap flow volumes throughout the summer, reaching a maximum of 46.6 L in September.

Notably, the differences in sap flow between the two irrigation methods became more pronounced from July to September. In the smart automatic irrigation method, the difference between sap flow volume and irrigation volume ranged from 0.3 to 0.6 L, while in the semi-automatic method, the difference was much higher, ranging from 8.4 to 45.7 L.

### 3.5. Correlation Between Sap Flow and Various Meteorological Variables Under Irrigation Treatments

The correlation between sap flow volumes of trees under different irrigation treatments and various meteorological variables, including soil temperature, soil moisture, air temperature, relative humidity, solar radiation, and wind speed, was analyzed. The results indicated that meteorological factors such as temperature, solar radiation, and humidity significantly impact sap flow.

**Figure 11:**
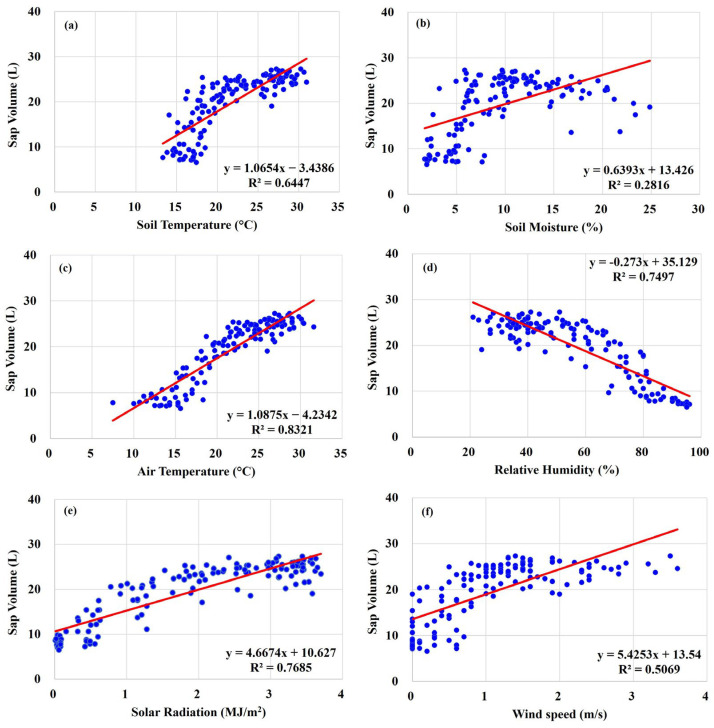
Relationship between sap flow volume in *Prunus* × *yedoensis* and weather parameters. (**a**) Sap flow versus soil temperature; (**b**) sap flow versus soil moisture; (**c**) sap flow versus air temperature; (**d**) sap flow versus relative humidity; (**e**) sap flow versus solar radiation; (**f**) sap flow versus wind speed.

In Figure 11a,e, air temperature and sap flow volume show a strong correlation (R^2^ = 0.8321), and solar radiation also exhibits a high correlation (R^2^ = 0.7685). Figure 11c,f demonstrate a positive correlation between soil temperature and sap flow volume (R^2^ = 0.6447), and wind speed was found to increase transpiration and sap flow as it increased (R^2^ = 0.5069). Conversely, Figure 11d shows that relative humidity had a negative correlation with sap flow (R^2^ = 0.7497). Meanwhile, the correlation between soil moisture and sap flow was relatively low (R^2^ = 0.2816).

### 3.6. Growth Characteristics of Seedlings Under Different Irrigation Treatments

**Figure 12:**
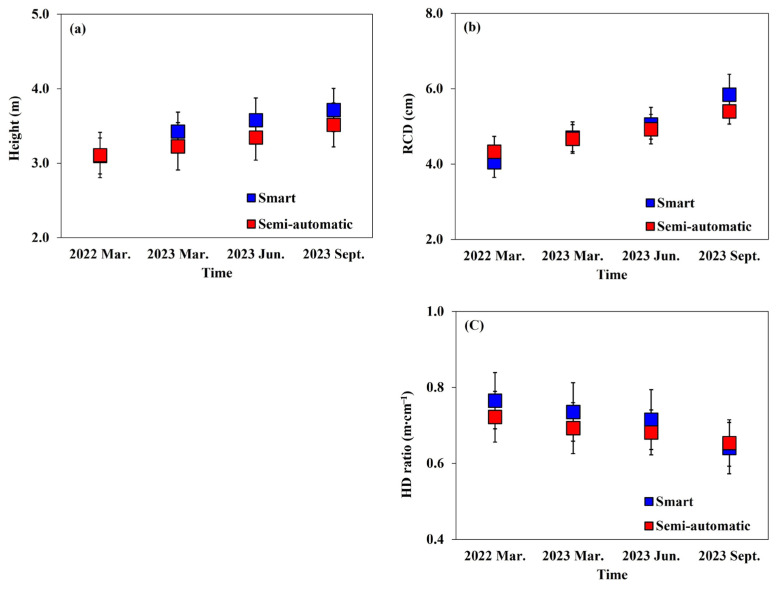
The growth patterns of height (**a**), root collar diameter (**b**), and H/D ratio (**c**) by different irrigation treatments of *Prunus* × *yedoensis* from March 2022 to September 2023. Repeated measures of ANOVA by Duncan’s multiple range test at 5% levels.

The growth and root collar diameter growth of *Prunus* × *yedoensis* trees varied significantly with different irrigation treatments (Table 3, Figure 12). Height growth (*p* < 0.01) and relative growth rate (*p* < 0.001) were higher in the smart automatic irrigation system. Similarly, root collar diameter growth (*p* < 0.001) and relative growth rate (*p* < 0.001) also tended to be higher in the same treatment group. The height-to-diameter ratio (H/D ratio) ranged from 0.64 to 0.65 m·cm^−1^ across all treatments, showing no significant differences between the irrigation methods

### 3.7. Changes in Photosynthesis Parameters Under Differnet Irrigation Treatments

The smart automatic irrigation treatment showed a 50.9% higher light compensation point compared to the semi-automatic irrigation treatment (34.2 ± 5.9 vs. 22.7 ± 8.1). The light saturation point was also higher in the smart automatic irrigation group. Additionally, the dark respiration values were greater under the smart automatic irrigation treatment (Table 4).

### 3.8. Chlorophyll Fluorescence Change Under Different Irrigation Treatments

**Figure 13:**
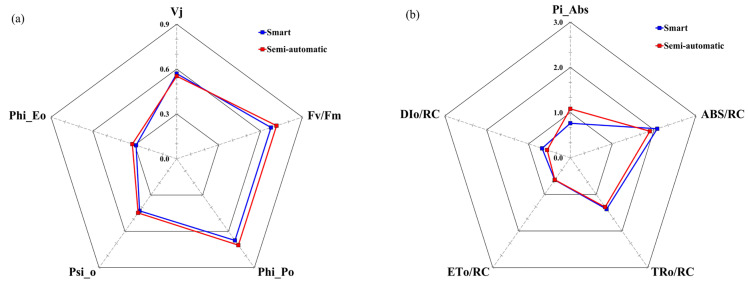
Chlorophyll fluorescence from March 2023 (**a**) to September 2023 (**b**) on containerized trees of *Prunus* × *yedoensis* grown under two different irrigation treatments. Repeated measures of ANOVA by Duncan’s multiple range test at 5% levels.

In the semi-automatic irrigation treatment, micro-variation fluorescence VJ was higher (Figure 13). The maximum quantum yield of the initial photochemical reaction, Phi_Po, and the efficiency of electron transport beyond QA-, Psi_O, Phi_Eo, also showed significant decreases in the semi-automatic irrigation group. Parameters indicating changes in energy flow per reaction center, such as ABS/RC, TRo/RC, and DIo/RC, increased in the semi-automatic irrigation treatment, whereas ETo/RC showed a slight decrease.

The maximum quantum yield of photosystem II in the dark-adapted state, Fv/Fm, decreased in the semi-automatic irrigation treatment (0.68) compared to the smart automatic irrigation treatment (0.72).

The PIABS value, which signifies the efficiency of energy conservation during the reduction of electron carriers utilizing absorbed light energy, decreased to 29.6% in the semi-automatic irrigation treatment compared to the smart automatic irrigation treatment.

## 4. Discussion

### 4.1. Changes in Seedling Characteristics by Irrigation Method

The sapwood area of *Prunus* × *yedoensis* varied significantly depending on the irrigation method, with the smart automatic irrigation area having a larger bark. Sapwood, as recently formed tissue, plays a crucial role in the upward movement of water absorbed from the roots. Despite being young trees, the fact that sapwood makes up more than 60% of the area suggests a significant impact on the movement of water and sap.

### 4.2. Changes in Soil Moisture Content by Weather Conditions and Irrigation Method

From March to September, the soil moisture content within containers was measured based on irrigation treatments. With the smart automatic irrigation method, moisture content maintained in the optimal levels according to prior research by the National Institute of Forest Science, South Korea, whereas it fell below the recommended standards in the semi-automatic irrigation method.

### 4.3. Changes in Flow Velocity and Sap Flow by Irrigation Treatment and Weather Conditions

Changes in flow velocity and sap flow were observed based on irrigation treatment and weather conditions. The study area’s climate varied monthly, with May being the driest month due to low rainfall and high vapor pressure deficit (VPD). These climatic changes are closely related to the plants’ water demand capacity. Rainfall during the monitoring period led to a decrease in global solar radiation and VPD, aligning with findings by Nadezhdina et al. [34].

Sap flow exhibited a pattern similar to temperature changes, gradually increasing after sunrise, peaking around 1 PM, and then gradually decreasing from 4 PM, forming a ‘bell-shaped’ curve. This pattern is likely due to the evaporative demand responding to changing atmospheric temperatures, reaching a peak in the early afternoon (12:00–14:00) and gradually decreasing to zero by midnight.

### 4.4. Changes in Flow Velocity and Sap Flow by Irrigation Treatment and Weather Conditions

Comparisons of flow velocity by irrigation treatment showed significantly higher velocities with smart automatic irrigation compared to semi-automatic irrigation. This result mirrors the pattern in soil moisture content; with smart automatic irrigation, smoother soil moisture supplies increased xylem flow velocity, thereby increasing sap flow speed and volume. Daily sap flow patterns were similar to those reported by many researchers for individual trees, with significantly higher flow rates observed during the day compared to nighttime water absorption. Loustau et al. [35] found that nighttime sap flow accounts for only 12% of daily total transpiration.

Sap flow mirrored temperature changes, leading to observations of decreased sap flow compared to the previous day across all irrigation treatments, likely due to stomatal closure caused by heat stress.

Sap flow volumes exhibited seasonal variations, influenced by environmental conditions such as temperature and relative humidity. During spring (March–May), both irrigation methods showed relatively low sap flow volumes, corresponding to lower temperatures and reduced plant water demand. The semi-automatic method recorded higher sap flow volumes, likely because the fixed irrigation schedule did not reflect real-time plant water needs, resulting in over-irrigation. In summer (June–September), sap flow volumes significantly increased, peaking in July with the smart automatic irrigation method. This suggests that smart automatic irrigation systems can meet real-time plant water demands and reduce unnecessary irrigation. Sap flux density is heavily influenced by environmental factors such as air temperature (Ta), photosynthetically active radiation (PAR), relative humidity (RH), vapor pressure deficit (VPD), and wind speed (WS) [36,37]. Additionally, sap flow changes with weather factors throughout different growth season periods [38].

The semi-automatic irrigation method consistently recorded higher sap flow volumes in summer, peaking in September, indicating that timer-based irrigation may lead to excess watering during high-demand periods like summer. The differences between the two methods were particularly pronounced from July to September, with smart automatic irrigation showing a 0.3–0.6 L difference between sap flow and irrigation volumes, while semi-automatic irrigation showed a much larger difference of 8.4–45.7 L. This highlights smart automatic irrigation as a relatively efficient water management method, which is capable of preventing excessive irrigation and efficiently utilizing water resources.

### 4.5. Relationship Between Tree Sap Flow, Meteorological Parameters, and Stem Water Potential

Tree sap flow volumes under different irrigation treatments were significantly influenced by meteorological factors such as temperature, solar radiation, and humidity. There was a strong correlation between temperature and sap flow volume, as well as between solar radiation and sap flow. This indicates that as temperature and solar radiation increase, transpiration activity rises, promoting sap flow. These findings align with correlations observed for *M. styphelioides*, where temperature (R^2^ = 0.7412) and solar radiation (R^2^ = 0.752) were similarly correlated with sap flow [22]. Thus, various meteorological factors show a positive correlation with sap flow volume, suggesting that increases in these factors can lead to increased sap flow.

Conversely, Figure 11d demonstrates a negative correlation between relative humidity and sap flow (R^2^ = 0.7497), indicating that lower humidity results in reduced atmospheric water potential, thereby increasing sap flow in trees. Meanwhile, the correlation between soil moisture and sap flow was relatively low (R^2^ = 0.2816), suggesting that soil moisture has a limited effect on sap flow.

### 4.6. Growth Characteristics of Seedlings Under Different Irrigation Treatments

The growth and root collar diameter growth of *Prunus* × *yedoensis* trees varied significantly with irrigation treatments. The smart automatic irrigation system resulted in superior height and root collar diameter growth, likely due to its impact on soil moisture content. Generally, tree growth is heavily influenced by moisture content in the growing medium, with a proportional relationship between tree growth rate and soil moisture levels. Poor moisture conditions result in lower growth rates, while adequate moisture leads to higher growth rates [39,40]. Herbaceous plants also show differences in stem growth based on water absorption or irrigation amounts [41,42].

In experiments with tropical species like *Eucalyptus pellita* and *Acacia mangium*, conducted with irrigation intervals of 1–3 days, treatments with irrigation daily showed the highest growth rates in height and root collar diameter. Conversely, longer irrigation intervals resulted in poorer growth [43]. Similarly, for tulip trees, the best height and root collar diameter growth was reported with once-daily irrigation [44]. These results, consistent with this study, indicate that irrigation frequency affects tree growth.

The H/D ratio, representing the ratio of height to root collar diameter, quantifies whether a tree is more stocky or spindly [45,46]. The H/D ratio ranged from 0.64 to 0.65 m·cm^−1^ across treatments, showing no significant differences between irrigation methods.

### 4.7. Changes in Photosynthetic Parameters Under Different Irrigation Treatments

The smart automatic irrigation treatment exhibited a 34.4% higher light compensation point compared to the semi-automatic irrigation treatment, suggesting it can more efficiently meet the minimum requirements for photosynthesis. The light saturation point was also higher in the smart automatic irrigation group, indicating greater effectiveness in maximizing photosynthetic efficiency. Additionally, higher dark respiration values in the smart automatic irrigation treatment suggest a tendency to consume energy consistently while maintaining photosynthetic activity. Notably, there were relatively larger differences in water use efficiency and maximum photosynthetic rate, indicating that smart automatic irrigation is effective in enhancing plant growth.

### 4.8. Changes in Chlorophyll Fluorescence Under Different Irrigation Treatments

Light energy absorbed by Photosystem II (PSII) antenna chlorophyll (ABS) excites the reaction center (P680), which then transfers electrons to nearby pheophytin through charge separation, reducing the initial electron acceptor QA (TRo). During this process, some energy fails to be captured by the reaction center and is released as heat or other forms (DIo). Following QA reduction, electrons are transported through a series of carriers (ETo) and finally reduce NADP+ to NADPH [47]. The JIP test effectively illustrates these energy transfer characteristics. The O-J phase reflects the imbalance between QA reduction and reoxidation at PSII’s reaction centers, while the I-P phase signifies rapid reduction of the plastoquinone pool (PO pool) [48].

In Figure 13, microvariation fluorescence VJ is higher in the semi-automatic irrigation treatment, indicating low reoxidation rates of QA- and electron transport interruption, which are linked to inactivation of the oxygen-evolving complex (OEC) [49]. Energy transfer ratios and fluorescence yields at each stage of photochemical reactions, such as Phi_Po, Psi_O, and Phi_Eo, showed significant decreases in the semi-automatic irrigation treatment.

Parameters indicating changes in energy flow per reaction center, such as ABS/RC, TRo/RC, and DIo/RC, increased in the semi-automatic treatment, while ETo/RC tended to decrease. The increase in ABS/RC suggests more reaction centers in a reduced state, indicating partial inactivation [50]. The increases in TRo/RC and DIo/RC imply that energy captured and released as heat from each reaction center has risen as overall active reaction center numbers have decreased. Particularly, the increase in DIo/RC is closely related to photoinhibition, understood as a protective mechanism against excess energy influx under water stress [51]. The decrease in ETo/RC indicates inhibited electron transport to reduce P700 in Photosystem I after QA reduction.

The maximum quantum yield of PSII in the dark-adapted state, Fv/Fm, decreased in the semi-automatic irrigation treatment (0.68) compared to the smart automatic treatment (0.72). Under stress, the maximum fluorescence value (Fm) decreases, and the ground state fluorescence value (Fo) increases. These values rise with higher chlorophyll content, and their reduction in the semi-automatic treatment suggests diminished energy transfer ratios and fluorescence yields at each photochemical reaction stage, indicating water stress due to drought [47].

PIABS reflects energy conservation efficiency during electron carrier reduction using absorbed light energy [52], encompassing three key features: the total density of active reaction centers, the proportion of energy absorbed by reaction centers captured for photochemical processes, and electron transport efficiency post-QA reduction. Known to be more sensitive than the maximum quantum yield of PSII (Fv/Fm) as an environmental stress indicator [47,49,53,54], PIABS is a good indicator for assessing and monitoring water stress, which is known to decrease PIABS [49,55]. In the semi-automatic irrigation treatment, PIABS decreased to 29.6% compared to the smart automatic irrigation treatment.

Overall, the semi-automatic irrigation treatment showed reduced energy capture for photochemical processes and increased non-utilized energy, leading to decreased PSII activity. This suggests a mechanism to increase reduced reaction centers and non-photochemical energy dissipation to prevent photodamage due to excessive energy caused by water stress under this irrigation treatment.

First, the smart automatic irrigation system enhances photosynthetic efficiency by achieving higher light compensation and saturation points. Second, it increases the maximum photosynthetic rate (Pn max) and water use efficiency (WUE), reducing unnecessary water supply and efficiently using water according to real-time moisture needs. Third, it efficiently increases sap flow during the day and responds better to various climatic conditions. Fourth, temperature and solar radiation have a significant impact on sap flow, while relative humidity shows a negative correlation. In conclusion, the smart automatic irrigation system minimizes water wastage and provides optimal moisture supply during high water demand periods like summer.

## 5. Conclusions

This study demonstrates that smart automatic irrigation systems are more effective than semi-automatic irrigation in water management and promoting plant growth. Smart automatic irrigation systems more efficiently meet the photosynthetic demands of plants and maximize photosynthetic efficiency, as evidenced by higher light compensation and saturation points. Additionally, smart automatic irrigation increases the maximum photosynthetic rate and water use efficiency, reducing unnecessary water supply and efficiently using water according to real-time moisture needs. It is more effective in increasing sap flow during the day and responds well to changing climatic conditions. Temperature and solar radiation significantly impact sap flow, while relative humidity shows a negative correlation. In conclusion, smart automatic irrigation systems reduce unnecessary water wastage during high-demand periods and provide optimal moisture supply, making them an important reference for developing sustainable agricultural and horticultural irrigation management strategies.

## Figures and Tables

**Table 1:** Equations and definitions of chlorophyll fluorescence parameters (modified from Gorbe et al. [33]).

Abbreviation	Mathematical	Description
Vj	(F_J_ − F_O_)/(F_M_ − F_O_)	Relative variable fluorescence at the J-step
Fv/Fm	1 − F_O_/F_M_	Maximum quantum yield of primary PSII photochemistry
Phi_Eo	ETo/ABS	Probability that an absorbed photon leads to electron transport further than QA
Phi_o	ETo/TRo	Probability that an absorbed photon leads to reduction of QA
Phi_Po	TRo/ABS	Probability that an absorbed photon leads to reduction further than QA
PI_ABS	(RC/ABS) (φP_O_/(1 − φP_O_)) (Ψ_O_/(1 − Ψ_O_))	Performance index for energy conservation from photons absorbed by PSII antenna, to the reduction of QB
ABS/RC	M_O_ (1/V_J_)(1/φP_O_)	Absorption flux per RC
TRO/RC	M_O_(1/V_J_)	Trapped energy flux per RC (at t = 0)
ETO/RC	(M_O_/V_J_)(1 − V_J_)	Electron transport flux from QA to QB per RC (at t = 0)
DI_O_/RC	(ABS/RC) − (TR_O_/RC)	Dissipated energy flux per RC (at t = 0)

**Table 2:** Wood properties of the *Prunus* × *yedoensis* used in these experiments.

Treatment	Bark Depth (mm)	Sapwood Depth (mm)	Heartwood Depth (mm)	Total Basal Area (TBA)		
				Area (cm^2^)	Treatment	Bark Depth (mm)
Smart automatic irrigation	1.4 ± 0.1	11.0 ± 0.6	8.2 ± 0.3	13.4 ± 0.8	9.5 ± 0.6	71.0 ± 0.1
Semi-automatic irrigation	1.6 ± 0.2	9.1 ± 0.5	8.5 ± 0.7	11.6 ± 1.5	7.4 ± 0.8	64.1 ± 2.4

**Table 3:** The root diameter, height, and H/D ratio of *Prunus* × *yedoensis* under different irrigation treatments.

Irrigation Treatment	Height (cm)		Root Collar Diameter (mm)		H/D (m·cm^−1^)
	Growth	Relative Growth Rate	Growth	Relative Growth Rate	
Smart automatic	3.71 ± 0.29 ^a^	0.0012 ± 0.0004 ^a^	5.84 ± 0.54 ^a^	0.0034 ± 0.0008 ^a^	0.64 ± 0.07 ^a^
Semi-automatic	3.52 ± 0.30 ^b^	0.0008 ± 0.0004 ^b^	5.40 ± 0.34 ^b^	0.0020 ± 0.0006 ^b^	0.65 ± 0.06 ^a^
*t*-value	−2.70 **	−4.43 ***	−3.76 ***	−7.16 ***	0.80 ^NS^

Mean within a column followed by the same letters are not significantly different at the 5% level by Duncan’s multiple range test. ^NS^ *p* ≥ 0.05, ** *p* < 0.01, *** *p* < 0.001.

**Table 4:** The photosynthesis parameters of *Prunus* × *yedoensis* under different irrigation treatments.

Irrigation Treatment	L_comp_ (µmol photon·m^−2^·s^−1^)	L_sat_ (µmol photon·m^−2^·s^−1^)	Rd (µmol CO_2_·m^−2^·s^−1^)	Pn max (µmol CO_2_·m^−2^·s^−1^)
Smart automatic	34.19 ± 5.85 ^a^	1661.98 ± 28.19 ^a^	5.84 ± 0.54 ^a^	0.0034 ± 0.0008 ^a^
Semi-automatic	22.66 ± 8.06 ^b^	1588.99 ± 77.80 ^a^	5.40 ± 0.34 ^b^	0.0020 ± 0.0006 ^b^
*t*-value	−2.39 *	−1.77 ^NS^	−2.85 *	−1.02 ^NS^
**Irrigation** **Treatment**	**AQY**	**E** **(mmol H_2_O·m^−2^·s^−1^)**	**gs** **(mmol H_2_O·m^−2^·s^−1^)**	**WUE** **(µmol CO_2_·mmol H_2_O^−1^)**
Smart automatic	0.06 ± 0.00 ^a^	1.52 ± 0.28 ^a^	0.06 ± 0.01 ^a^	8.07 ± 0.96 ^a^
Semi-automatic	0.05 ± 0.00 ^a^	1.39 ± 0.33 ^a^	0.05 ± 0.01 ^a^	4.12 ± 0.46 ^b^
*t*-value	−3.35 *	0.64 ^NS^	−1.49 ^NS^	−8.17 ***

Mean within a column followed by the same letters are not significantly different at the 5% level by Duncan’s multiple range test. ^NS^ *p* ≥ 0.05, * *p* < 0.05, *** *p* < 0.001. L_comp_ (light compensation point), L_sat_ (light saturation point), Rd (dark respiration), Pn max (maximum photosynthesis rate), AQY (net apparent quantum yield), E (stomatal transpiration rate), gs (stomatal conductance), WUE (water use efficiency). Repeated measures of ANOVA by Duncan’s multiple range test at 5% levels.

## Data Availability

The data presented in this study are available upon request from the corresponding author.
